# Immunosuppressive landscape in hepatocellular carcinoma revealed by single-cell sequencing

**DOI:** 10.3389/fimmu.2022.950536

**Published:** 2022-07-28

**Authors:** Yi Bai, Dapeng Chen, Chuanliang Cheng, Zhongmin Li, Hao Chi, Yuliang Zhang, Xiaoyu Zhang, Shaohai Tang, Qiang Zhao, Bing Ang, Yamin Zhang

**Affiliations:** ^1^ Department of Hepatobiliary Surgery, Tianjin First Central Hospital, School of Medicine, Nankai University, Tianjin, China; ^2^ Tianjin First Central Hospital Clinic Institute, Tianjin Medical University, Tianjin, China; ^3^ Tianjin First Central Hospital Clinic Institute, School of Medicine, Nankai University, Tianjin, China; ^4^ College of Life Sciences, Nankai University, Tianjin, China; ^5^ Oncology Department, Tianjin First Central Hospital, School of Medicine, Nankai University, Tianjin, China

**Keywords:** primary liver cancer, hepatocellular carcinoma, single cell sequencing, tumor immune microenvironment, prognosis

## Abstract

**Background/Aims:**

Hepatocellular carcinoma (HCC), accounting for 75-85% of primary liver cancer cases, is the third leading cause of cancer-related death worldwide. The purpose of this research was to examine the tumor immune microenvironment (TIME) in HCC.

**Methods:**

We investigated the HCC TIME by integrated analysis of single-cell and bulk-tissue sequencing data to reveal the landscape of major immune cell types.

**Results:**

Regulatory T(Treg) cells were found to be specifically distributed in the TIME of HCC. Several immune checkpoints, including TNFRSF4, TIGIT and CTLA4, were found to be uniquely overexpressed in Treg cells, and the glycolysis/gluconeogenesis pathway was enriched in Treg cells. We also discovered the presence of two NK-cell subsets with different cytotoxic capacities, one in an activated state with antitumor effects and another with an exhausted status. In addition, memory B cells in HCC were found to exist in a unique state, with high proliferation, low differentiation, and low activity, which was induced by overexpression of PRAP1 and activation of the MIF-CD74 axis.

**Conclusions:**

We revealed the TIME landscape in HCC, highlighting the heterogeneity of major immune cell types and their potential mechanisms in the formation of an immunosuppressive environment. Hence, blocking the formation of the TIME could be a useful therapeutic strategy for HCC.

## Introduction

Primary liver cancer is the sixth most commonly diagnosed cancer and the third leading cause of cancer death worldwide (1). Hepatocellular carcinoma (HCC), accounting for 75-85% of primary liver cancer cases, is the largest source of cancer-related disease burden. Specifically, approximately 70-80% of patients with HCC are diagnosed at an advanced stage (2), when curative therapies, including surgical resection, radiofrequency ablation, and liver transplantation, are not feasible. In addition, as 80~90% of HCC patients have concomitant cirrhosis, and the application of different therapeutic options might be limited because of the patient’s overall health status. Patients with advanced HCC typically receive multimodal therapy primarily comprising targeted therapy and immunotherapy (3).

Most tumors are complex and develop and evolve under robust selective pressure from their microenvironment, which includes nutrition-related, metabolic, immunological, and therapeutic components. Such pressure promotes the diversification of both malignant and nonmalignant compartments of the tumor microenvironment (TME), resulting in a degree of intratumoral heterogeneity (ITH) that enables aggressive disease progression and resistance to treatment (4). Multiple studies have proven that the immune component within the tumor, known as the tumor immune microenvironment (TIME), which includes immune cells, extracellular immune factors, and cell surface molecules, is closely associated with tumor development, recurrence, and metastasis (5, 6). The recognition of the importance of the TIME has given rise to another treatment option—immune checkpoint inhibitors (ICIs)—which have revolutionized cancer therapy. Blocking antibodies targeting immune inhibitory receptors such as CTLA-4, PD-1, and PD-L1 are by far the most extensively utilized immunotherapeutic drugs, with promising curative effects in a variety of cancers (7). Nivolumab, the first anti-PD1 monoclonal antibody approved as second-line therapy for HCC, has an objective response rate (ORR) of 15-20% and a disease control rate (DCR) of 58-64% (8). However, the ORR of tremelimumab (which targets CTLA-4) is only 7.2% (9). Thus, the intricate immune microenvironment of HCC needs to be explored, and doing so may improve the understanding of the effects of immunotherapy. Furthermore, the discovery of new therapeutic targets could pave the way for new treatments.

Different from traditional bulk RNA sequencing, single-cell RNA sequencing (scRNA-seq) has enabled researchers to investigate intratumoral heterogeneity at the single-cell level (10, 11). Thanks to the application of scRNA-seq, the field of single-cell genomics has massively expanded over the past few years, revealing surprising new insights into tissue biology and disease causes. Therefore, in this study, we performed a single-cell transcriptome analysis of seven HCC tumor tissue and paired normal tissue samples, thoroughly exploring the components and effector functions of major immune cell types and discovering novel biomarkers that might be employed as new therapeutic targets.

## Materials and methods

### Data acquisition and preparation

The Cancer Genome Atlas (TCGA) database was used to obtain the bulk gene expression profiles of HCC samples as well as the corresponding clinical data. There were 374 tumor samples and 50 paracancerous samples in the TCGA liver hepatocellular carcinoma (LIHC) cohort. Three fibrous lamellar carcinomas and seven mixed carcinomas in the 374 tumor samples were removed. In addition, samples from the same patients were removed. As a result, we ended up with 360 tumor samples. The scRNA-seq count matrix was downloaded from the GSE149614 dataset, which contains 21 samples, including 10 primary tumor samples, 8 corresponding peritumor liver samples, 1 metastatic lymph node sample and 2 portal vein tumor thrombus samples. Because our study focused on the heterogeneity between tumor and normal tissues, we selected corresponding tumor and normal samples. Since sample HCC07T only had data for 510 cells, HCC07T and the equivalent HCC07N sample were removed. Ultimately, a total of seven tumor samples and corresponding peritumor tissues were included in our study.

### Single-cell analysis

The Seurat R package (12) was used for quality control (QC). Cells were removed when (a) RNA counts were fewer than 50; and (b) mitochondrial gene expression percentages were more than 5%. Data normalization was performed using the NormalizeData function in Seurat. The top 14 principal components and the top 1500 variable genes were chosen for the subsequent analysis. Then, cell clusters were detected by using Seurat’s FindClusters function (resolution = 0.5) and displayed *via* 2D t-distributed stochastic neighbor embedding (tSNE) (13). Cells from each cluster were compared to the annotated reference dataset using the SingleR package (14). According to the comparison results and the identified cell markers, each cluster was annotated. To reveal the differentiation of cell groups, we used Monocle2 (15), an R package built for pseudotime analysis. Furthermore, we estimated cell differentiation with online analysis *via* CytoTRACE (CytoTRACE (stanford.edu)), which is a robust approach for predicting cell differentiation with scRNA-seq data.

### Functional enrichment analysis

Differentially expressed genes (DEGs) were selected by the FindMarker function in Seurat, and genes with adjusted p values < 0.05 were considered for subsequent analysis. Subsequently, Gene Ontology (GO) pathway enrichment analysis was performed utilizing clusterProfiler R packages (16). In this study, the gene set enrichment of each cell cluster was determined by gene set enrichment analysis (GSEA). Gene sets and KEGG pathways were obtained from MSigDB. Then, gene set variation analysis (GSVA) with 50 hallmark gene sets was performed among cell clusters. Furthermore, we used the AUCell R package to estimate the score of customized gene sets across cell groups.

### InferCNV analysis

The InferCNV R package was developed to infer the copy number variation (CNV) of tumor cells based on the scRNA-seq matrix. Human genetic coordinate information was downloaded from https://data.broadinstitute.org/Trinity/CTAT/cnv/. Hepatocytes from normal tissue were considered the reference set, and the CNV of hepatoma cells was calculated. The final heatmap was generated after denoising.

### SCENIC analysis

The SCENIC R package (17) is designed to assess gene regulatory networks and can guide the identification of transcription factors (TFs) and cell states. The GENIC3 method was used to extrapolate coexpression modules between transcription factors and candidate target genes. Each module, i.e., each regulon, contains a TF and its target genes. The cisTarget human motif database was utilized to enrich the gene signature, and targets in this signature were pruned according to the default set of cis-regulatory cues. Then, we assessed the activity of each regulon in each cell using the AUCell algorithm. The ComplexHeatmap and heatmap R packages were used for visualization.

### Cell–cell communication

To study the cell group-to-cell group interactions, we employed CellPhoneDB (18), which can be used to analyze cell-to-cell communication at the molecular level. The receptor–ligand pairs with p <0.05 between each cell group were preserved, and we removed the receptor–ligand pairs within the same cell type. Finally, the number of receptor–ligand pairs was counted and visualized by Cytoscape.

### Construction of a hepatoma cell differentiation trajectory

To verify the significance of genes related to cancer differentiation trajectory, we generated a hepatoma cell differentiation trajectory using the Monocle2 R package. Using the ConsensusClusterPlus package in R, 360 HCC samples in the TCGA LIHC cohort were divided into 3 clusters based on their expression of genes related to hepatoma cell evolution. Then, Kaplan–Meier (K-M) survival curves for each cluster were generated. Correlation analysis of clinical characteristics was performed for the 3 clusters.

### Statistical analysis

Analyses between two groups were performed utilizing the Wilcoxon test. One-way ANOVA was used to compare three or more groups. Generally, statistical analyses were conducted by R studio (version 4.1.1) and GraphPad Software (version 8.0). The significance level was set at P<0.05.

## Results

### Single-cell transcriptome analysis identified cell compositions

**Figure 1:**
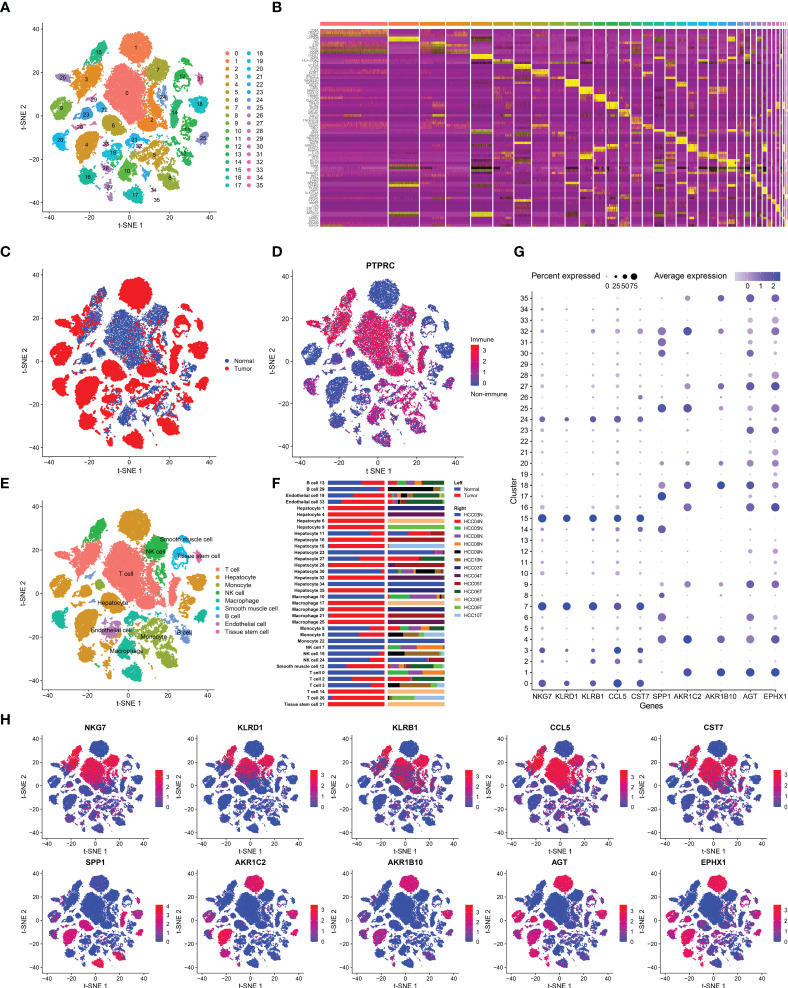
Cell-type classification in HCC. **(A)** t-SNE plot of 36 cell clusters. **(B)** Heatmap of the top five marker genes in each cell cluster. **(C)** Cell distribution in tumor and normal tissues. **(D)** Cell distribution in immune cells (PTPRC^+^) or nonimmune cells (PTPRC^-^). **(E)** t-SNE plot exhibiting the cell types in HCC. The labels were automatically annotated by SingleR. **(F)** Bar plot of the distribution of all identified cell clusters: the proportion of each cell in tumor and normal samples (left) and in each patient (right). **(G)** The top five DEGs with the highest expression in tumor and normal tissues. **(H)** t-SNE plot of the top five DEGs.

Seven HCC samples and corresponding normal tissues were included in our study. A total of 39,667 cells passed QC, of which 21,121 were from tumor tissues and the rest were from normal tissues. These cells were then grouped into 36 clusters (**Figure 1A**), with the top five most significant genes highlighted and plotted on a heatmap (**Figure 1B**). Nine major cell types were identified in HCC. In addition to hepatocytes, smooth muscle cells, endothelial cells, and tissue stem cells, there were many immune cells (PRPTC^+^), including T cells, monocytes, natural killer (NK) cells, macrophages, and B cells (**Figures 1D**, **E**). When investigating the distributions of all 9 cell lineages, we noticed that the main cell type in tumor tissue was hepatoma cells, while in normal tissue, it was T cells (**Figure 1C**). Interestingly, there was an extreme difference in T cells and NK cells between intratumor and peritumor tissues. We believe that the depletion of T cells and NK cells in tumor tissue is associated with the formation of immunosuppression and is inextricably linked to tumor progression. In contrast to those of T and NK cells, the proportions of macrophages and monocytes were increased in HCC tumor tissues (**Figure 1F**). Neoantigens are tumor-specific antigens (TSAs) derived from the expression of mutated genes in cancer cells and are not present in normal tissues. These neoantigens may attract macrophages as well as DCs differentiated from monocytes that can engulf and present them, leading to the aggregation of macrophages and monocytes in HCC tumor tissues.

Subsequently, we analyzed DEGs between tumor and normal tissues (**Table S1**) and selected the top five DEGs with the highest expression, which were SPP1, AKR1C2, AKR1B10, AGT, and EPHX1 in tumor tissues and NKG7, KLRD1, KLRB1, CCL5, and CST7 in normal tissues. The expression of the above genes was then marked in each cell lineage (**Figures 1G**, **H**). The highly expressed genes in normal tissues were mainly distributed in T and NK cells. NKG7, KLRD1, and KLRB1 are marker genes of T cells and NK cells, which is in line with the fact that the proportions of T and NK cells in tumor samples were significantly smaller than those in paracancerous tissues. In addition, EXPH1, AKR1C2 and AKRB10 were predominantly expressed in hepatoma cells. EPHX1 was first purified from the liver and is engaged in a variety of physiological activities, including lipid metabolism and detoxification of heterologous substances (19). Furthermore, it has been demonstrated that EPHX1 causes resistance to 5-fluorouracil in hepatoma cells and promotes chemoresistance in leukemia (20, 21). AKR1C1 and AKRB10 are both members of the human aldo-keto reductase family. Multiple studies have revealed that AKR1C1 is upregulated in various cancers, such as lung, breast and cervical cancers, and is associated with cancer metastasis and chemotherapy resistance (22, 23). Moreover, emerging experiments have also identified the role of AKR1B10 in HCC invasion and drug resistance (24). We validated that AKR1C1, AKRB10 and EXPH1 are promising biomarkers of HCC and potential therapeutic targets by scRNA-seq analysis.

### Treg cells were enriched in HCC and had distinct metabolic characteristics

**Figure 2:**
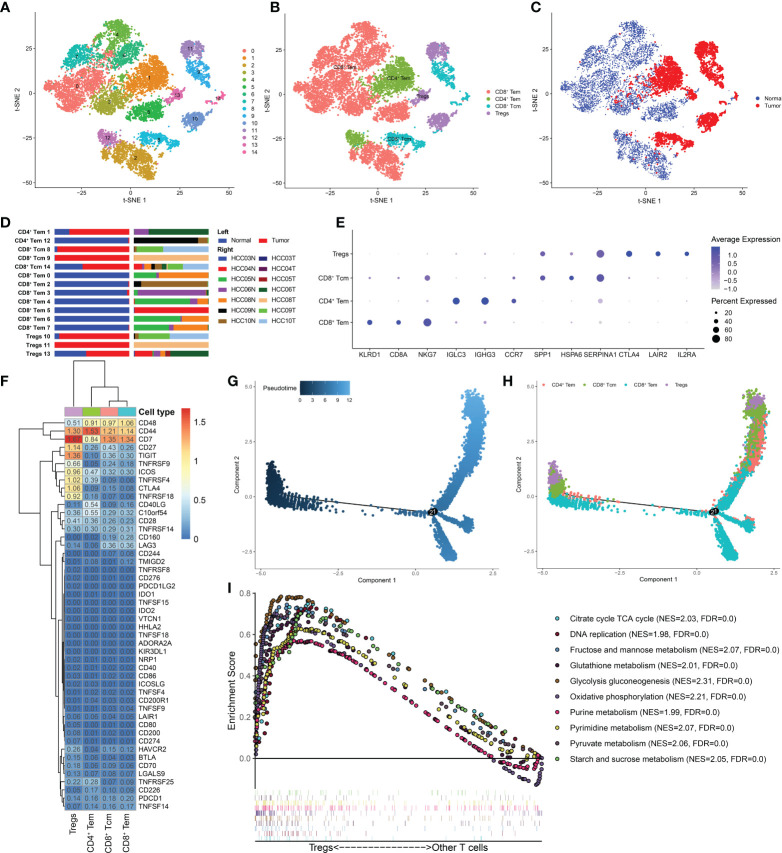
Treg cells were enriched in HCC and had distinct metabolic characteristics. **(A)** t-SNE plot of 15 subclusters of T cells and **(B)** four major T-cell subtypes: CD8^+^ Tem, CD4^+^ Tem, CD8^+^ Tcm, and Treg cells. **(C)** Distribution of the four T-cell subtypes in tumor and normal samples and **(D)** in each patient. **(E)** Top five marker genes of the four T-cell subtypes. **(F)** Heatmap of immune checkpoint expression in the four T-cell subtypes. The row Z score represents the expression level. A differentiation trajectory of T cells, colored based on pseudotime **(G)** and cell type **(H)**, is shown. **(I)** GSEA revealed that metabolic pathways were significantly enriched (FDR < 0.05) in Treg cells.

T cells have been found to be associated with prognosis in a variety of malignancies. During the past two decades, immunotherapies targeting T cells, including chimeric antigen receptor (CAR) T-cell therapy, adoptively transferred tumor-infiltrating lymphocytes, and immune checkpoint inhibitor (ICI) therapies, have been found to be effective in suppressing cancer growth (25). However, T cells have high heterogeneity, and different subtypes play varying effector functions within the TME. To investigate the heterogeneity of T cells, we clustered 12884 T cells into 15 subgroups, mainly four cell subtypes: CD8^+^Tem, CD4^+^Tem, CD8^+^Tcm, and Treg cells (**Figures 2A**, **B**). Among them, CD8^+^ Tem cells, which showed the highest proportion, were found in normal tissues, while CD4^+^ Tem, CD8^+^ Tcm, Treg cells were mainly found in HCC tissues (**Figures 2C**, **D**). This difference in distribution is likely related to the distinct functions of these T-cell subtypes: CD8^+^ Tem cells mostly reside in peripheral tissues and lymphoid tissues and play an effector role (26); CD4^+^ Tem cells travel to the infection site to exert killing functions (27), while Treg cells reside in tumor tissue and play an immunosuppressive role (28). The top three marker genes of the four T-cell subtypes are shown in **Figure 2E**.

Next, we determined the expression levels of immune checkpoints in the four T-cell subtypes (**Figure 2F**). Several inhibitory checkpoints, such as TIGIT, CTLA4, TNFRSF4 and TNFRSF9, were only overexpressed in Treg cells. Despite being a minor component of immune cells, Treg cells can play a critical role in the TIME network. Treg cells suppress anticancer immunity, which hinders protective immunosurveillance of cancer and prevents the formation of an effective antitumor immune response, thereby promoting tumor development and progression. The immune checkpoints mentioned above that are only highly expressed in Treg cells can be considered potential therapeutic targets to restore immunity against cancer cells in HCC. Furthermore, we mapped the T cell differentiation trajectory by utilizing the Monocle2 R package (**Figures 2G**, **H**). Notably, Treg cells from HCC tissues were mainly found at the beginning and end of the differentiation trajectory, while CD8^+^ Tem cells were mainly found in the middle of the differentiation trajectory. Recent research on Treg cells has shown that targeting Treg cells in the end-stage of differentiation is an effective strategy to stimulate antitumor immunity (28). Strategies to deplete and control Treg cells by modulating Treg cell differentiation and development deserves further exploration.

In addition, the GSEA results showed that compared with other T-cell subtypes, Treg cells showed enrichment of metabolic pathways, such as glycolysis, gluconeogenesis, glutathione metabolism, and starch and sucrose metabolism (**Figure 2I**), indicating that Treg cells in HCC are not metabolically inhibited and have metabolic flexibility. Emerging studies have highlighted the critical role of metabolism in immune cells. Treg cells can proliferate with tumor development, maintaining their immunosuppressive effects because of increased metabolism in the TME. Tumors can also support Treg cell immunity by modulating metabolites, resulting in tumor immune escape (29, 30). In recent years, targeting metabolism has become a strategy to inhibit tumor development, and strategies employing DCs and macrophages have made progress (31). However, there is still much unknown about the connection between Treg cell metabolic pathways and the epigenetic control of gene expression. Our research will contribute to a better understanding of the tumor-promoting mechanism of aberrant Treg cell metabolism, as well as its effects on the differentiation and development processes.

### Macrophages are related to the immunosuppressive environment in HCC

**Figure 3:**
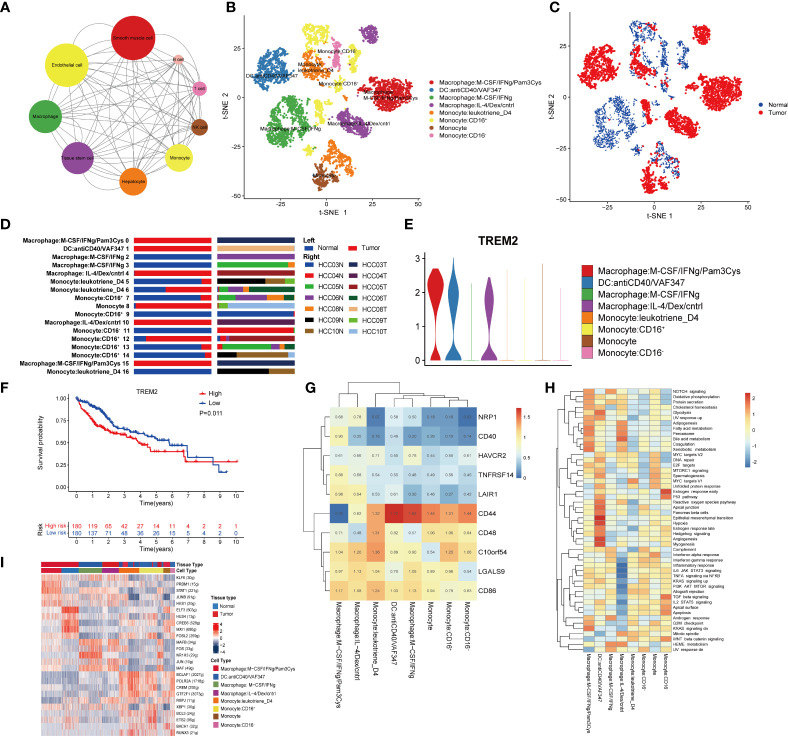
Macrophages are related to the immunosuppressive environment of the TME. **(A)** Interaction network constructed by CellPhoneDB. The size of the circles represents the interaction count. A larger size means more interaction with other cell types. **(B)** t-SNE plot of the eight subtypes of macrophages. **(C)** Distribution of the eight subtypes of macrophages in tumor and normal samples and **(D)** in each patient. **(E)** Violin plots of the expression of TREM2 in the eight subtypes of macrophages. **(F)** Kaplan–Meier survival curve for patients in the TCGA LIHC cohort. A log rank p value <0.05 was considered statistically significant. **(G)** Heatmap of immune checkpoint expression in macrophages. The row Z score represents the expression level. **(H)** Differences in hallmark pathway activities scored with GSVA. The t values calculated by a linear model are shown. **(I)** Heatmap of the AUC scores of the expression of transcription factors identified by SCENIC.

To study the interaction network in the HCC microenvironment, the Python-based cell–cell communication molecular analysis tool CellphoneDB was used to identify ligand–receptor pairs in the TME. Cytoscape was used to visualize the resulting network. Unexpectedly, except for epithelial cells and smooth muscle cells in HCC tissues, macrophages had the richest communication with other cell types (**Figure 3A**), revealing that macrophages play a significant role in the TME.

To assess the heterogeneity of macrophages, we clustered all macrophages into eight subclusters (**Figure 3B**). The phenotypes of clusters 1 to 8 are depicted in **Figure 3B**: macrophages: M−CSF/IFNγ/Pam3Cy; DCs: anti-CD40/VAF347; macrophages: M−CSF/IFNγ; macrophages: IL−4/Dex/cntrl; monocytes: leukotriene D4; monocytes: CD16^+^, monocytes; and monocytes: CD16^−^. By annotating the cell sources, we discovered that clusters 1, 2, 4, and 7 were derived from only cancer tissues, while clusters 3 and 8 were derived from only normal tissues. In addition, clusters 5 and 6 were derived from both cancer and normal tissues (**Figures 3C**, **D**). Therefore, cluster 3 was identified as a macrophage phenotype from normal tissues and thus was employed as a control. According to the expression of IFNγ and IL-4, cluster 1 was considered closer to M1 macrophages, and cluster 4 was considered closer to M2 macrophages. Although we could not accurately annotate the mononuclear and DC subsets, their functions should not be ignored.

As accumulating evidence has revealed a role of TREM2 in tumor-associated macrophages (TAMs) and myeloid-derived suppressor cells (MDSCs), we analyzed the expression of TREM2 in the eight clusters and found it to be high in cluster 1, cluster 2 and cluster 4 (**Figure 3E**), which were all derived from cancer tissues. TREM2 has been linked to poor prognosis and is a vital in inducing immunosuppression in the TME (32–36). To further study the clinical value of TREM2, we divided 360 HCC patients from the TCGA LIHC cohort into two groups according to the expression level of TREM2. Interestingly, the TREM2-overexpression group had a significantly worse prognosis (**Figure 3F**), consistent with previous research results, suggesting that TREM2 may be important for immunosuppression in the TME.

Subsequently, the expression of immune checkpoints among the 8 clusters was determined. Clusters 1 and 4 had higher expression levels of LAIR1 than the other clusters (**Figure 3G**). LAIR1 has been found to be associated with tumor immunosuppression and has recently been reported to block the LAIR1 and TGF-β signaling pathways to remodel the TME, making PD-L1-mediated tumor eradication possible (37).Our results also suggest that PD-L1-mediated tumor eradication could be a potential therapeutic strategy.

GSVA showed that the adipogenesis, fatty acid metabolism and bile acid metabolism pathways were enriched in cells from cluster 4, while the inflammatory response and the complement and interferon (IFN) pathways, which are the hallmarks of M2-like TAMs (as described in a previous study), were significantly suppressed (**Figure 3H**). It is worth mentioning that DCs were more significantly enriched in processes closely related to tumor metabolism, such as epithelial-mesenchymal transition, hypoxia, and angiogenesis.

Finally, we analyzed the expression of TFs in each subgroup. We found that compared to other clusters, cluster 1 and cluster 4 had higher expression of KLF6, PRDM1, STAT1, JUNB, and HES1 (**Figure 3I**). Researchers have reported that KLF6 plays an essential role in immunosuppression and modulates neutrophil maturation (38–42), while PRDM1 is a crucial epigenetic gene associated with T-cell terminal differentiation (43, 44). In other words, targeting PRDM1 would enable the generation of superior antitumor T cells. Furthermore, the IRF3/STAT1 pathway was reported to be associated with M2 polarization in a murine model of sarcoma (45). All of these findings indicate the vital role of macrophages in immunosuppression in the TME, as well as the potential mechanisms and TFs involved in this process.

### Two distinct NK-cell subsets with different cytotoxic capacities in HCC

**Figure 4:**
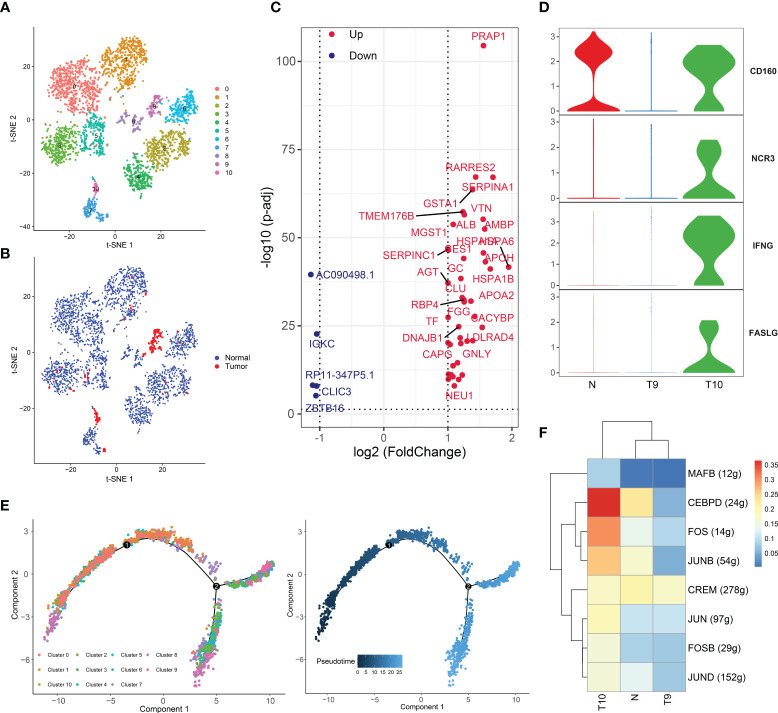
Two distinct NK-cell subsets with different cytotoxic abilities in HCC. **(A)** t-SNE plot of the eleven subsets of NK cells. **(B)** Distribution of the eleven NK-cell subtypes in tumor and normal samples. **(C)** Volcano plot of the differentially expressed genes (DEGs) between tumor-derived NK cells and normal tissue-derived NK cells. The upregulated genes (log2(fold change) >1) are colored red, while the downregulated genes (log2(fold change) less than -1) are colored blue. Upregulated and downregulated genes are annotated. **(D)** Violin plots of the expression of several activating receptors, including CD160, NCR3, IFNG (IFNγ) and FASLG, in cluster 9, cluster 10 and normal tissues. **(E)** Differentiation trajectory of NK cells, colored for pseudotime (right) and cell subset (left). **(F)** Heatmap of the AUC scores of the expression of transcription factors identified by SCENIC.

NK cells have recently been a hotspot in cancer immunology research. We detected 3151 and 187 NK cells in peritumor and tumor tissues, respectively, and clustered them into 11 subsets (**Figures 4A**, **B**). As previously described, the proportion of NK cells was dramatically decreased in tumor tissues compared to normal tissues. NK cells can kill cancer cells without MHC restriction and do not require preactivation by DCs or antibodies. NK cells play a critical role in the immunosurveillance protecting against cancer (46). The obvious decrease in NK cells in HCC suggests that cancer cells shape an environment that inhibits NK-cell proliferation, possibly by hypoxia or metabolic inhibition, which directly leads to resistance to attacks from NK cells.

DEGs of NK cells were identified between tumor and normal tissues (**Figure 4C**). Notably, HSP70 (including HSPA1A and HSPA1AB), a ubiquitous molecular chaperone, was highly expressed in tumor-derived NK cells. The major action of HSP70 is to maintain protein homeostasis and to mediate cytoprotective effects. Under adverse stress conditions, HSP70 can improve cell resistance to the environment and acts as a protector against stress (47). Tumor-resident NK cells are in a state of stress in the TME, and the antitumor effect of these NK cells can be supported by maintenance of hemostasis induced by upregulated HSP70. In addition, SERPINA1, which regulates hydrolase activity, and CLU, which stabilizes proteins, were upregulated (48, 49).

Interestingly, tumor-derived NK cells were distributed in clusters 9 and 10, while NK cells of other clusters came from normal tissues (**Figures 4A**, **B**). To investigate the heterogeneity of cluster 9 and cluster 10, we detected the expression of activating receptors of NK cells, including CD160, NCR3, IFNG (IFNγ), and FASLG, in cluster 9, cluster 10, and normal tissues (**Figure 4D**). CD160 potentiates NK-cell activation and cytotoxicity and induces the secretion of the cytokines IFN-γ, IL-6, IL-8 and TNF-α. The FasL protein is encoded by FASLG and is secreted by activated NK cells (50). After the ligand binds with the corresponding receptor on cancer cells, the apoptotic system of cancer cells is initiated. Notably, these genes were upregulated in cells from cluster 10 and normal tissue. Thus, compared to the cells of cluster 9, the cells of cluster 10 has more potent cytotoxicity, and the cells from cluster 9 exhibited an exhausted status. Compelling evidence has suggested that NK cells become exhausted in the presence of tumors and chronic infections, displaying low cytotoxicity and effector function (51). NK-cell exhaustion is also observed in HCC (52), yet the exact regulatory mechanisms have been poorly explored. Hence, we performed a pseudotime analysis (**Figure 4E**). It was found that cluster 10 cells were present at the beginning of the differentiation trajectory, while cluster 9 cells, which represented an exhausted NK-cell subset, existed at the end of the trajectory. We believe that cluster 10 cells gradually transform into cluster 9 cells with tumor infiltration and TME formation.

Subsequently, SCENIC analysis showed that the genes regulated by CEBPD, FOS, and JUN were significantly activated in cluster 10 (**Figure 4F**). JUN/FOS can regulate NK-cell immune activity through IF-2 (53), and CEBPD can promote NK-cell development and immunity (54). All of these findings confirmed the presence of two distinct NK-cell clusters with different cytotoxic capacities in HCC and suggest that the TME may gradually develop several mechanisms to suppress the immunotoxicity of NK cells. We revealed candidate TFs involved in this process, and the mechanisms of these TFs in NK cells are worth exploring. Furthermore, these TFs may serve as therapeutic targets.

### Low activity state of memory B cells in HCC

**Figure 5:**
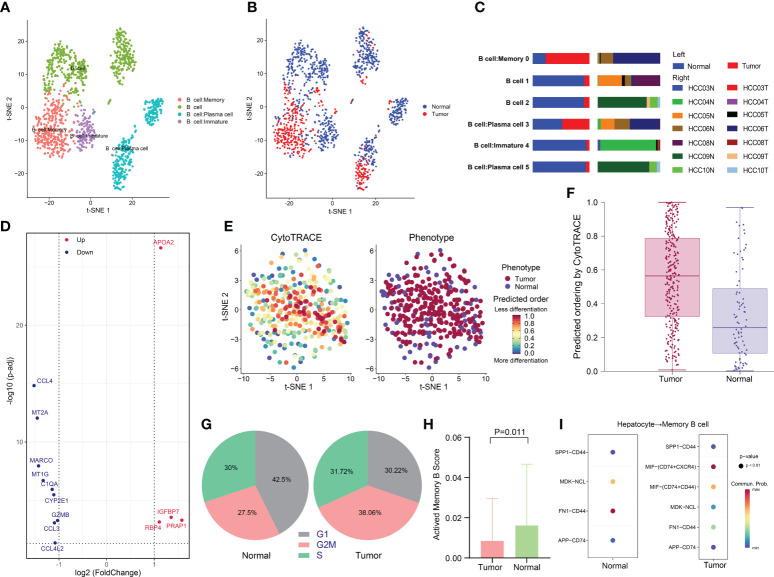
Low activity state of memory B cells in HCC. **(A)** t-SNE plot of the four B-cell subtypes in HCC. **(B)** Distribution of the four B-cell subtypes in tumor and normal samples and **(C)** in each patient. **(D)** Volcano plot of the DEGs between tumor-derived memory B cells and normal tissue-derived memory B cells. The upregulated genes (log2(fold change) >1) are colored red, while the downregulated genes (log2(fold change) less than -1) are colored blue. Upregulated and downregulated genes are annotated. (E and F) Differentiation status of different tissue-derived memory B cells as determined using CytoTRACE. A higher score indicates a lower degree of differentiation. **(G)** Pie charts of the proportions of cells in each stage of the cell cycle in different tissue-derived memory B cells. **(H)** Bar plot of the activated memory B-cell signature scores of memory B cells in tumor and normal samples. The signature scores were calculated based on the expression of activated memory B-cell-related genes, including CD86, AICDA, DHR59, EBI3, TBX21, KLF10, ZEB2, TFEC, ZBTB32, YBX3, CXCR3, ITGAX, and SIGLEC6. **(I)** Receptor–ligand pairs between hepatocytes and memory B cells.

A total of 1395 B cells were ultimately obtained after data processing and screening, and they were categorized into six clusters and annotated into four subtypes: B cells, immature B cells, memory B cells and plasma cells (**Figures S1A** and **5A**). Notably, we noticed that almost all immature B cells were derived from normal tissues, while memory B cells were derived from tumor tissues (**Figures 5B**, **C**).

Previous investigations have shown a strong infiltration of memory B cells in HCC, suggesting a specific immune response to the tumor (55–57). The prognostic impacts of memory B cells in HCC remain controversial. Memory B cells have been shown to have tumor-killing potential, with their presence in the HCC microenvironment indicating a good prognosis (58). However, other studies have reported that B cells restrain the antitumor response in several ways (59). Therefore, we conducted further analysis of memory B-cell subsets. The DEGs of memory B-cell subsets between tumor and normal tissues were identified (**Figure 5D**). Strikingly, we observed that PRAP1 was significantly upregulated in memory B cells derived from cancer tissue. PRAP1 has been demonstrated to be a novel p53 target gene that promotes cancer cell resistance to chemotherapy drugs such as 5-fluorouracil (5-FU) by cell cycle arrest to protect cells from apoptosis and contribute to cancer cell survival (60). In addition, PRAP1 can downregulate mitotic arrest deficient 1 (MAD1), which is a key factor in mitotic checkpoint signaling, leading to chromosomal instability and promoting the occurrence of HCC (61).

Next, we compared the differentiation potential of different tissue-derived memory B cells. Using CytoTRACE, we predicted a higher differentiation potential for tumor-derived memory B cells (**Figures 5E**, **F**). Similarly, a comparison of the cell cycles of the different tissue-derived memory B cell subtypes found that a larger proportion of tumor-derived memory B cells were in the G2/M phase, suggesting a more robust proliferative capacity, consistent with the low differentiation status of tumor-derived memory B cells (**Figure 5G**). To investigate functional heterogeneity, we assessed the expression of a gene set related to memory B-cell activation to predict the functional status (62). The results showed that there were significantly fewer memory B cells in the activated state in tumor tissues than in peritumor tissue (p = 0.011), which may be linked to the occurrence of an antitumor response (**Figure 5H**).

Given the crucial role of the crosstalk between the TME and cancer cells in tumorigenesis and progression, we compared receptor–ligand pairs between cancer cells and memory B cells. In particular, we discovered that macrophage migration inhibitory factor (MIF), which interacts with CD74^+^CXCR4^+^ and CD74^+^CD44^+^, was specifically expressed in tumor samples (**Figure 5I**). MIF has been confirmed to contribute to a variety of facets of tumor growth, including cell proliferation, differentiation, and angiogenesis. MIF can bind to its receptor CD74 in the TME, which is present on TAMs, DCs, Treg cells, and MDSCs, facilitating immunological escape and cancer growth (63, 64). For the first time, our study found that MIF may inhibit memory B-cell activity in HCC and that inhibiting the MIF-CD74 axis may be a new treatment strategy.

### Hypermetabolism and immunosuppression in hepatoma cells

**Figure 6:**
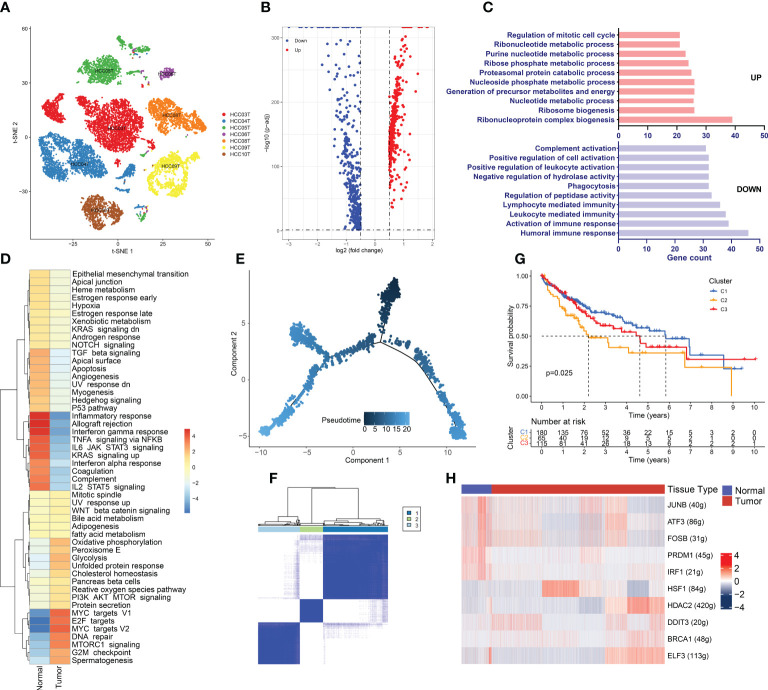
Hypermetabolism and immunosuppression in hepatoma Cells. **(A)** Cancer cell distribution across all tumor samples. **(B)** Volcano plot of the DEGs between tumor-derived hepatoma cells and normal tissue-derived hepatocytes. The upregulated genes (log2(fold change) >0.5) are colored red, while the downregulated genes (log2(fold change) less than 0.5) are colored blue. **(C)** Gene Ontology analysis of the DEGs. The upregulated and downregulated DEGs are annotated. FDR <0.05 was considered significantly enriched. **(D)** Differences in hallmark pathway activities scored with GSVA. The t values calculated by a linear model are shown. **(E)** Differentiation trajectory of cancer cells colored for pseudotime. **(F)** TCGA LIHC patients were clustered into 3 clusters by ConsensusClusterPlus based on the expression of genes related to the cancer cell evolution states. **(G)** Kaplan–Meier survival curves for the patients in the 3 clusters. A log rank p value < 0.05 was considered statistically significant. **(H)** Heatmap of the AUC scores of the expression of transcription factors identified by SCENIC.

Hepatoma cells are derived from hepatocytes, so we compared CNV between hepatoma cells and normal hepatocytes. InferCNV analysis showed obvious CNV in hepatoma cells (**Figure S2A**). Meanwhile, hepatoma cells expressed high levels of APOA2, APOA1, AMBP, TTR, APOH, and ASGR1, which is consistent with what was previously observed by Sun Y et al. in HCC (**Figure S2B**) (65). Furthermore, hepatoma cells originating from the same patient tended to cluster together (**Figure 6A**), which indicated that there was significant heterogeneity between cancer cells across different patients. Heterogeneity is likely to lead to different responses to the same treatment among patients. In addition, multiple cancer cell subsets were discovered in the same lesion, revealing the existence of different tumor cell subtypes within tumor tissue (**Figure S2C**). Different subtypes of tumor cells exhibit differences in immune characteristics, growth rate, and invasive ability, resulting in different sensitivities to antitumor drugs.

The DEGs between hepatoma cells and hepatocytes were detected by scRNA-seq data analysis, which can avoid the interference of other cells in the TME (**Table S2** and **Figure 6B**). Strikingly, members of the metallothionein family, including MT1M, MT1H, MT1G, MT1F, MT1E, MT1X, and MT1A, were significantly downregulated in cancer cells. Metallothionein, a low-molecular-weight metal-binding protein, plays key roles in a range of biological processes in the human body, including participating in metal ion homeostasis and detoxification, regulating cell growth and proliferation, modulating immune inflammatory responses, and protecting the body from DNA damage and oxidative stress (66). Accumulating studies have shown the vital functions of MT1 proteins in tumor growth, invasion, and immune escape in kidney, breast, lung, and ovarian cancers (67). Recent studies have shown decreased MT1 expression in HCC (68). Promoter methylation can lead to significant repression of MT1G and MT1M expression. Moreover, MT1G and MT1M promoter methylation was found to be associated with an increased incidence of vascular invasion or metastasis (69). Therefore, MT1 proteins can serve as biomarkers of HCC, and these findings may lead to the development of new and effective therapeutic modalities if the mechanism and function of MT1 proteins in the development of HCC can be elucidated.

GO enrichment analysis revealed that the upregulated DEGs were mainly enriched in metabolism-related terms, such as ribonucleotide metabolic processes, purine nucleotide metabolic processes, precursor metabolites, and energy production (**Figure 6C**). This result indicates the presence of a hypermetabolic state in hepatoma cells. Tumor initiation and progression require metabolic reprogramming of cancer cells to alter their metabolic pathways to meet the biological needs of metabolism and biosynthesis. Therefore, high metabolism is a distinctive feature of tumor cells and promotes proliferation and invasion. However, immune functions were found to be suppressed in cancer cells. This is in accordance with our previous analysis showing that the microenvironment of HCC is immune-suppressed. The GSVA results were generally in line with the GO enrichment analysis results (**Figure 6D**), and we observed that the inflammatory response, as well as the interferon α-response and interferon γ-response pathways, were significantly repressed in cancer cells. The Mtorc1 and MYC signaling pathways, which lead to high metabolism and proliferation of cancer cells, were considerably enriched. Enrichment of the G2/M checkpoint and DNA repair pathways indicated the presence of considerable DNA damage in cancer cells. SCENIC analysis showed that heat shock factor 1 (HSF1), which maintains proteostasis in response to stress environments by inducing the expression of heat shock proteins, was significantly upregulated in cancer cells (**Figure 6H**). Recent studies have demonstrated the roles of HSF1 in tumorigenesis, such as inhibiting apoptosis, reprogramming metabolism, and regulating the TME (70, 71). In addition, DDIT3, a stress-induced TF, controls genes involved in cell cycle arrest and/or apoptosis as its primary function. A recent study discovered the role of DDIT3 in balancing glycolysis and oxidative phosphorylation during glutamine deprivation in cancer cells (72). Both of the above TFs involved in stress regulation have been found to be overexpressed in hepatoma cells and to be associated with poor prognosis (**Figure S2D**). In the HCC TME, DDIT3 and HSP1 may together regulate apoptosis and metabolism to promote tumor cell proliferation and tumor progression. Therefore, HSF1 and DDIT3 can serve as biomarkers of clinical prognosis and promising drug targets.

Cancer cells have multiple subgroups with varying degrees of differentiation that can exhibit differences in various aspects. To stratify HCC patients based on the degree of differentiation of cells and provide precise treatment recommendations, we generated a cancer cell differentiation trajectory using the Monocle2 R package (**Figure 6E**). Markers of HCC stem cells, including CD44, EPCAM, and THYI (CD90), were expressed at the beginning of the cancer cell differentiation trajectory (**Figure S2E**). DEGs were selected based on the different cancer cell differentiation states, which influence tumor cell differentiation and progression, as well as patient prognosis (**Table S3**). Therefore, we divided TCGA LIHC patients into 3 clusters based on these DEGs using ConsensusClusterPlus (**Figure 6F**). The K–M analysis illustrated that the patients in the cluster 2 group had considerably shorter OS than those in the cluster 1 and cluster 3 groups (p<0.05) (**Figure 6G**). When we analyzed the clinical features of the three clusters, we found that grade, T stage, and stage were significantly different among the patients in the three groups (**Figure S2F**). The cluster 2 group had a larger number of patients with advanced stage disease and tumor hypofractionation, indicating that cancer cells with varying degrees of differentiation have diverse invasive capacities that influence cancer progression.

## Discussion

There remains a lack of treatment for HCC patients in the terminal stage who have missed the chance for surgery. Only a few patients can benefit from immunotherapy targeting immune inhibitory receptors of T cells, and some of them may develop drug resistance and stop responding to therapy. The TIME is the most dominant component of the TME and is critical for cancer progression as well as drug resistance. Immunosuppression is a characteristic of cancer, and crosstalk between cancer cells and immune cells ultimately leads to an environment that leaves patients with a weakened defense and often a worse prognosis (73). Because the TIME consists of a variety of immune cells and cytokines that frequently interact with each other, targeting one type of immune cell may lead to a series of changes in other microenvironments and relevant pathways. Combination therapies targeting several immune cells may thus be particularly effective in cancer treatment. Recent research has shown that the anti-tumor effects of Regorafenib, a multi-targeted tyrosine kinase inhibitor, are highly depended on its anti-tumor angiogenesis and anti-immunosuppressive properties, such as decrease of the TAM infiltration and enhancement of NK-cell cytolytic activity (74). Current research on the HCC TIME mainly focuses on T cells and macrophages, with few studies assessing the TIME as a whole. In this study, we investigated the TIME of HCC using single-cell sequencing and revealed the landscape of major immune cell types. The distribution and pathways of major cell subsets were analyzed, and some potential TIME-regulating mechanisms were discovered. The results of this study improve our understanding of the mechanisms of TIME formation and provide new ideas for immunotherapy.

Since the HCC TIME includes multiple immune cells and cytokines, we analyzed the predominant immune cell types in this study. We discovered a large number of Treg cells enriched in cancer tissue. Treg cells, the key component of immune homeostasis, maintain self-tolerance and suppress anticancer immunity. The presence of Treg cells is linked to tumor progression, aggressiveness, and metastasis. Their regulatory functions involve a wide range of immune cells other than T cells, including macrophages, DCs, neutrophils, NK cells, and innate lymphocytes. In addition to the typical expression of CD25, CD4, and FOXP3, Treg cells express a number of chemokine receptors and surface molecules, such as CTLA4, PD-1, and TIGIT, which are linked to anti-tumor immunity and may make them a direct target for ICI therapy. T-cell exhaustion, which is connected to tumor immune evasion, can be brought on by the overexpression of co-inhibitory receptors such CTLA4 and PD-1 (75). It has been reported that anti-CTLA4 and anti-PD1 monoclonal antibodies can deplete Treg cells and achieve clinical benefit. However, no other Treg cell-targeting therapies have been clinically proven to be effective, largely due to the difficulties associated with selectively targeting Treg cells (76). Our study revealed that, in addition to CTLA4, Treg cells uniquely overexpressed several immune checkpoints. Targeting these checkpoints may selectively deplete Treg cells, enabling precision immunotherapy. In addition, the glycolysis/gluconeogenesis pathway was enriched in Treg cells. Tumor cell metabolism is mainly dependent on glycolysis, which results in a high-lactate environment. Cancer cells can reprogram the metabolic pathways of Treg cells by communicating with them to induce adaptation to a high-lactate environment. Effector T cells are suppressed, while Treg cells are capable of surviving and proliferating within the TME; these Treg cells in turn support the formation of the TME, promoting immune escape of tumor cells and triggering tumor progression. Thus, altering the glycolytic pathway of Treg cells may induce activation of the entire immune system.

We also identified the presence of two NK-cell subsets with different activation statuses, one in an activated state with cancer-killing activity and another with exhaustion. The differentiation trajectory of NK cells in HCC was plotted using pseudotime analysis. The results showed a gradual depletion of NK-cell activity during TME formation. Recently, individuals with metastatic melanoma, NSCLC, and other tumor types have benefited significantly from ICIs. Strong T-cell anti-tumor immune responses are the basis of ICIs. Blocking NK cell-specific checkpoint receptors to reverse TME-induced NK cell exhaustion can revive NK cells’ direct cytotoxic activity against tumors and further initiate and enhance T cell-mediated adaptive anti-tumor immunity, according to a growing body of evidence (77). NK cell-based therapies are an effective complement to T cell therapy. Studies have found that combinations of anti-NKG2A monoclonal antibodies with anti-PD-1 monoclonal antibodies have showed promising outcomes in treating patients with advanced solid tumors (78). In this study, we observed the gradual functional depletion of NK-cell during the formation of TME by scRNA-seq analysis, and hindering this process is a new strategy for immunotherapy. In addition, the key TFs involved in this process was revealed, reversing the decreased expression of these TFs may restore the activity of NK cells.

Furthermore, memory B cells are enriched in tumor tissue, as observed by Tang B et al (56). However, the prognostic impacts of memory B cells in HCC remain controversial (58, 59). For the first time, our research found that memory B cells from HCC are in a unique state, with high proliferation, low differentiation, and low activity. Memory B cells are driven into this state by not only their own high expression of PRAP1 but also the influences of cancer cells *via* the MIF-CD74 axis. Currently, a detailed assessment of memory B cells in HCC has not been carried out, and our study suggests that the unique functional state and precise regulation of memory B cells may produce a good prognosis for patients.

Macrophages are at the center of cell–cell communication in the HCC TIME, and the prevailing perspective is that TAMs can be classified into M1-like (proinflammatory) and M2-like (anti-inflammatory) phenotypes. Our study suggests that the simple classification of M1-like and M2-like TAMs does not account for the complexity of TAMs. Some TAM subsets feature both M1-like and M2-like characteristics, and these subsets cannot be distinguished using conventional M1 and/or M2 macrophage markers. However, we discovered that TREM2 is uniquely expressed in tumor-derived DCs and macrophage subsets. Mounting evidence has revealed an immunosuppressive role of TREM2 in cancer (79, 80). TREM2 overexpression was associated with worse prognosis in the TCGA LIHC cohort. In addition, we revealed altered pathways and immune checkpoints in TAMs, which may provide new ideas for TAM-related immunotherapy.

Finally, using pseudotime analysis, we generated a hepatoma cell differentiation trajectory. For the first time, patients in the TCGA LIHC cohort were clustered into three distinct clusters based on their expression of differentiation-related genes. Notably, cluster 2 patients showed worse prognosis. Cancer cells in different differentiation stages exhibit differences in growth rate and invasive ability, resulting in different sensitivities to antitumor drugs. Therefore, individualized and precise treatment based on genetic testing will achieve increased clinical benefits.

In this study, we revealed the landscape of the TIME in HCC, highlighting the heterogeneity of major immune cell types and their potential mechanisms in the formation of an immunosuppressive environment. In summary, our research provides a novel theoretical basis for modulating the TIME in HCC and will aid the development of new immunotherapies.

## Data availability statement

The datasets presented in this study can be found in online repositories. The names of the repository/repositories and accession number(s) can be found in the article/**Supplementary Material**.

## Author contributions

YB designed the research scheme and perform the bioinformatics analyzes. SHT downloaded and organized the gene expression matrix, clinical information in HCC. DPC performed the statistical analyzes. YB and DPC wrote the manuscript. QZ and YMZ critically revised the article for essential intellectual content and provided administrative support. All authors read and approved the final version of the manuscript. All authors reviewed and revised the manuscript. QZ and YMZ were the guarantors for this study.

## Funding

This work was supported by the Tianjin Natural Science Foundation (20JCYBJC01310 and 21JCYBJC00320), the Tianjin Science and technology project (19ZXDBSY00010), and the Tianjin Health Science and technology project (TJWJ2021ZD002 and ZC20218).

## Acknowledgments

We thank Yanlong Zhang for assistance with the statistical methods.

## Conflict of interest

The authors declare that the research was conducted in the absence of any commercial or financial relationships that could be construed as a potential conflict of interest.

## Publisher’s note

All claims expressed in this article are solely those of the authors and do not necessarily represent those of their affiliated organizations, or those of the publisher, the editors and the reviewers. Any product that may be evaluated in this article, or claim that may be made by its manufacturer, is not guaranteed or endorsed by the publisher.

## Supplementary material

Supplementary Figure 1 t-SNE plot of the six subclusters of B cells

Supplementary Figure 2
**(A)** CNV heatmaps with hierarchical clustering from the InferCNV analysis. The reference cells are normal tissue-derived hepatocytes, and the test cells are tumor-derived hepatoma cells. **(B)** t-SNE plot of the six markers of HCC, APOA2, APOA1, AMBP, TTR, APOH, and ASGR1. **(C)** t-SNE plot of the twenty subclusters of hepatoma cells. **(D)** Kaplan–Meier survival analysis of patients in the TCGA cohort. **(E)** Differentiation trajectory of cancer cells colored based on gene expression. **(F)** The proportions of patients with different T stages, stages, and grades in each cluster. A p value <0.05 was considered statistically significant.

Supplementary Table 1 DEGs between tumor and normal tissues identified by Seurat’s FindClusters function

Supplementary Table 2 DEGs between hepatoma cells and normal hepatocytes

Supplementary Table 3 Genes related to hepatoma cell evolution based on pseudotime analysis

## Abbreviations

CNV, copy number variation; DEG, differentially expressed genes; DCR, disease control rate; GSEA, gene set enrichment analysis; GSVA, gene set variation analysis; HCC, Hepatocellular carcinoma; ICIs, immune checkpoint inhibitors; ITH, intratumoral heterogeneity; MIF, macrophage migration inhibitory factor; MDSCs, myeloid-derived suppressor cells; ORR, objective response rate; QC, quality control; tSNE, t-Distributed Stochastic Neighbor Embedding; TIME, tumor immune microenvironment; TAMs, tumor-associated macrophages; TSAs, tumor-specific antigens.
