# Supplementary figures and images for: Immunosuppressive landscape in hepatocellular carcinoma revealed by single-cell sequencing
Source: Front Immunol. 2022 Jul 28;13:950536. doi: 10.3389/fimmu.2022.950536 (PMC9365996; doi:10.3389/fimmu.2022.950536)

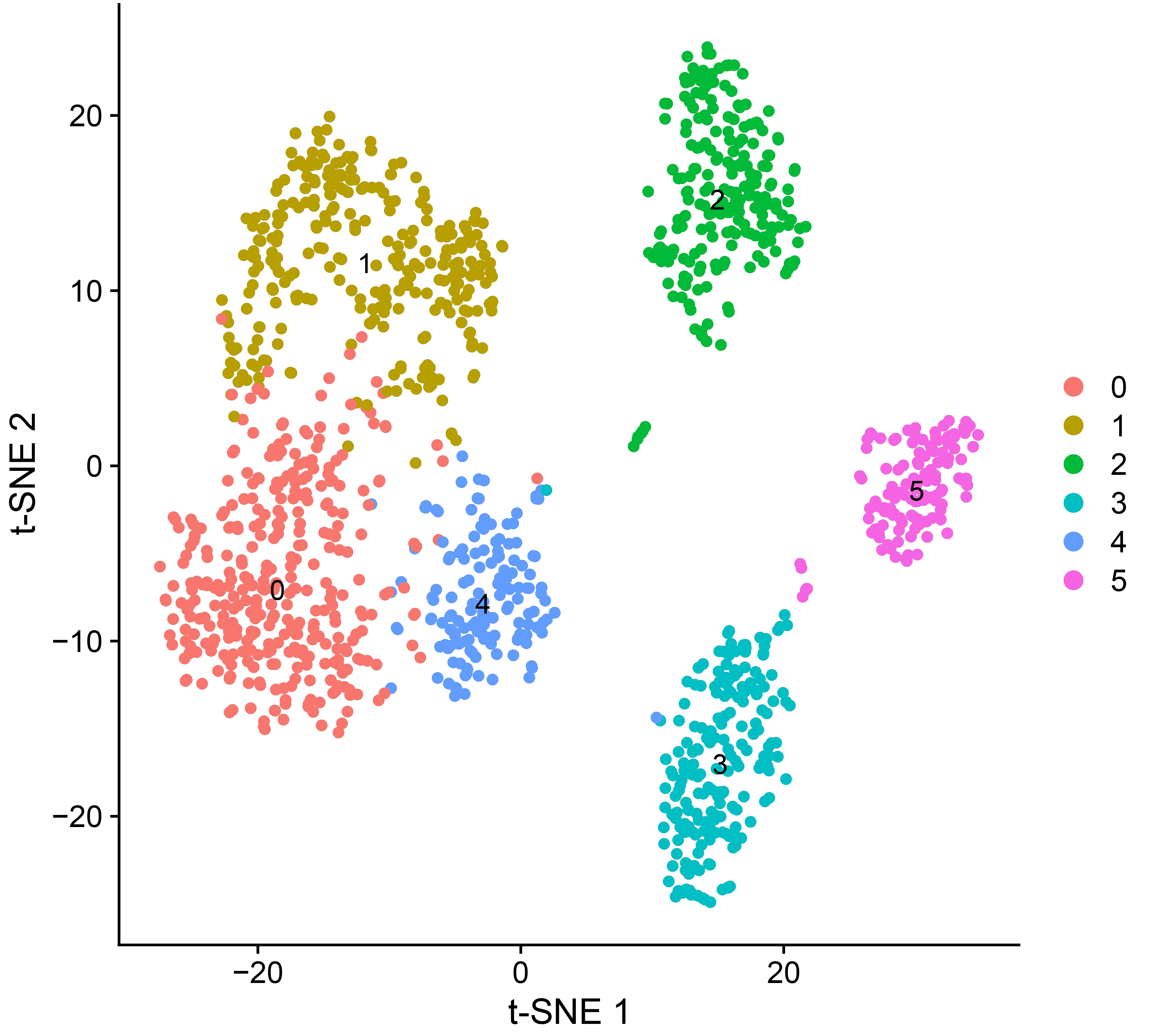

Supplement: Supplementary Figure 1 — t-SNE plot of the six subclusters of B cells [file Image_1.tif]

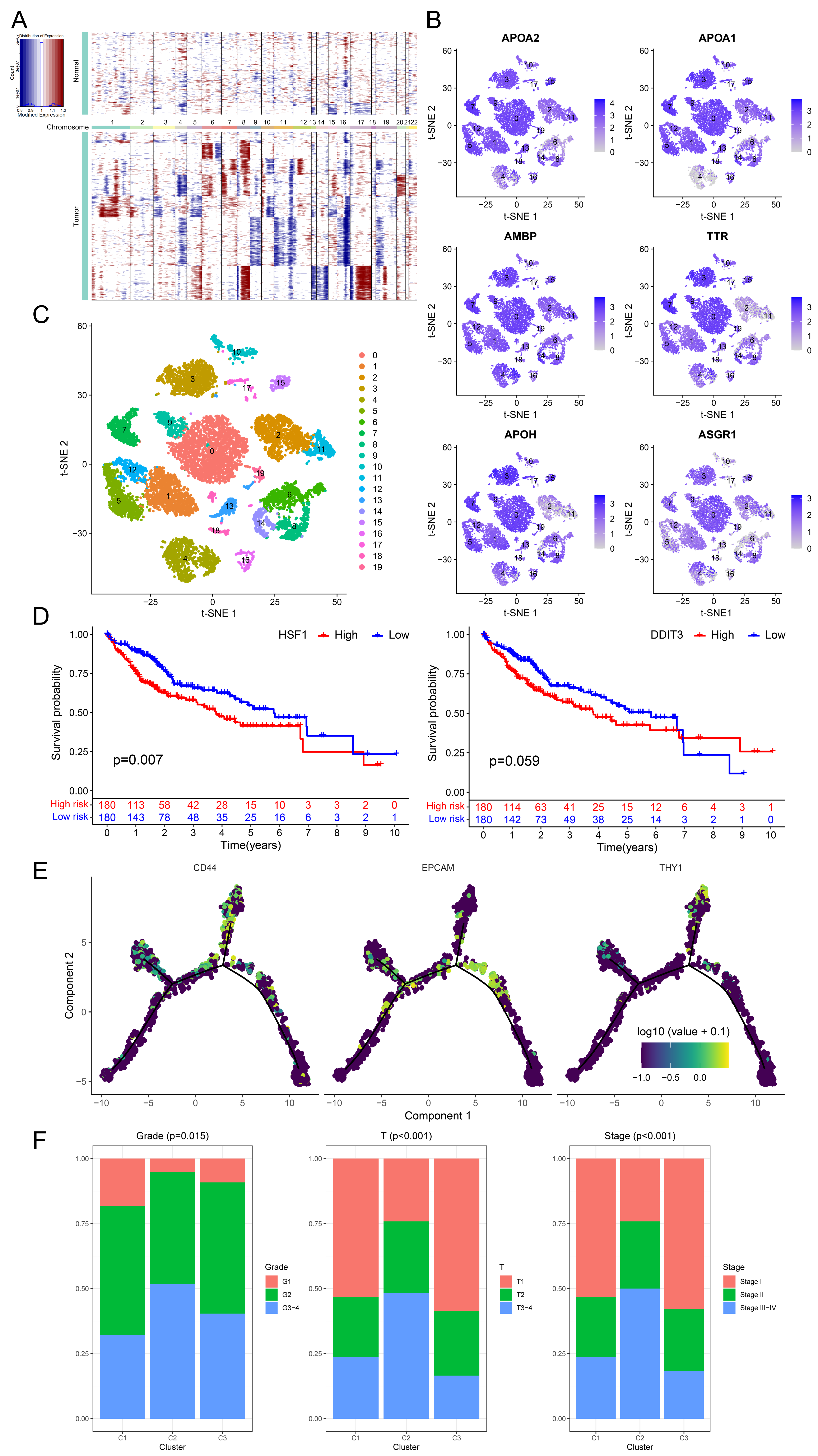

Supplement: Supplementary Figure 2 — (A) CNV heatmaps with hierarchical clustering from the InferCNV analysis. The reference cells are normal tissue-derived hepatocytes, and the test cells are tumor-derived hepatoma cells. (B) t-SNE plot of the six markers of HCC, APOA2, APOA1, AMBP, TTR, APOH, and ASGR1. (C) t-SNE plot of the twenty subclusters of hepatoma cells. (D) Kaplan–Meier survival analysis of patients in the TCGA cohort. (E) Differentiation trajectory of cancer cells colored based on gene expression. (F) The proportions of patients with different T stages, stages, and grades in each cluster. A p value <0.05 was considered statistically significant. [file Image_2.tif]
